# Development of Biodegradable Films Loaded with Phages with Antilisterial Properties

**DOI:** 10.3390/polym13030327

**Published:** 2021-01-20

**Authors:** Carol López de Dicastillo, Laura Settier-Ramírez, Rafael Gavara, Pilar Hernández-Muñoz, Gracia López Carballo

**Affiliations:** 1 Center of Innovation in Packaging (LABEN), Center for the Development of Nanoscience and Nanotechnology (CEDENNA), Department of Science and Food Technology, Faculty of Technology, Universidad de Santiago de Chile (USACH), Santiago 9170201, Chile; analopez.dediscastillo@usach.cl; 2 Packaging Lab., Institute of Agrochemistry and Food Technology, IATA-CSIC, Av. Agustín Escardino 7, 46980 Paterna, Valencia, Spain; laura.settier@iata.csic.es (L.S.-R.); rgavara@iata.csic.es (R.G.); phernan@iata.csic.es (P.H.-M.)

**Keywords:** bateriophage, *Listeria monocytogenes*, antimicrobial films, polyvinyl alcohol, sodium caseinate, sodium alginate

## Abstract

The inhibitory and bactericidal capacity of Listex P100 bacteriophage has been studied against different concentrations of *Listeria monocytogenes* in stationary and exponential phases. Three different matrices were employed to developed films incorporating Listex P100: (1) sodium caseinate, (2) sodium alginate mixed with gelatin, and (3) polyvinyl alcohol (PVOH). All the films were successfully developed by casting at room temperature. These active biodegradable films were optical, structural, and thermally characterized, and their antimicrobial capacities against *L. monocytogenes* were studied. The incorporation of phages did not affect the morphology, colour, opacity, and thermal stability of polymers. The antimicrobial analysis revealed the bacteriophage presented a high antimicrobial capacity against *L. monocytogenes* in the stationary phase (4.40 and 6.19 log reduction values or bactericide effect depending on the initial inoculum of the pathogen). Developed films showed antimicrobial capacity close to 1 log after 24 h of incubation at 30 °C. The effectiveness of PVOH films was greater under refrigeration conditions, reaching 2 log reduction after eight days of incubation. The use of these films as a coating in a food or as part of a packaging could improve food safety against the growth of pathogenic microorganisms such as *Listeria monocytogenes*.

## 1. Introduction

*Listeria monocytogenes* is a ubiquitous pathogen microorganism associated with a number of serious foodborne outbreaks. This Gram-positive foodborne pathogenic bacterium is responsible for the listeriosis disease. The manifestations of this disease usually produce symptoms similar to flu, but it can also generate septicaemia, meningitis, or meningoencephalitis and encephalitis [1]. Listeriosis can present dangerous consequences, especially to pregnant woman, newborn, children, elderly, and immunosuppressed people. Therefore, it is relevant to design control mechanisms to avoid the growth of this pathogen in food products. The design of active food packaging materials with antimicrobial capacity has been a strategy to reduce microbial incidence [2]. Several antimicrobial substances, such as metallic and metal oxide compounds, natural extracts, and essential oils, have been incorporated into different polymeric matrices, but the use of bacteriophages as active compounds has been undoubtedly an innovative alternative during recent years [3].

Bacteriophages (phages) are viruses able to infect and kill bacteria with high specificity. The main advantage of these viruses is their ability to attack specific harmful pathogens from food products without affecting the beneficial bacteria responsible for food organoleptic properties [4,5]. Because bacteriophages do not interact with other microorganisms or eukaryotic cells, they do not produce illness in animals and humans. Nowadays, there are some commercial bacteriophages such as Listex P100, which is a culture of safe and natural bacteriophages approved by the FDA (U.S. Food and Drug Administration) with a broad antibacterial spectrum towards *L. monocytogenes* strains.

Bacteriophages have great potential in the food industry as antimicrobial agents applied directly into food products or through their incorporation into the food packaging material for a more controlled release [6]. In this sense, phages can be inserted into polymeric materials and applied as active antimicrobial coatings or films. However, this method involves several complications due to the high sensitivity to desiccation [7]. Leung et al. (2018) have preserved bacteriophages by developing coatings comprised of pullulan and trehalose [5]. Common drying methods during films processing and storage humidity conditions are the two factors with the most relevant impact on the long-term antimicrobial activity of these active compounds [8]. Nevertheless, several developments based on the incorporation of phages into biopolymers with antimicrobial purposes have been successfully carried out. Phages incorporation on alginate films has been applied to prevent meat spoilage caused by *Pseudomonas fluoerescens* [9]. Cellulose acetate films containing bacteriophages have also shown antimicrobial activity against *Salmonella* Typhimurium [10]. On the other side, gelatin edible films incorporating bacteriophages against *Staphylococcus aureus* were tested in cheese; however, the best performance was obtained through direct immersion into film forming solution containing bacteriophages [11].

The antimicrobial effect of bacteriophage can be increased in combination with other antimicrobial agents. A phage cocktail with cinnamaldehyde on sodium alginate emulsion-based films has been studied against *Escherichia coli* and *Salmonella* Enteritidis, obtaining a synergic antimicrobial effect [12].

It must be considered that the choice of natural active agents is as relevant as the election of eco-friendly matrices in the development of active materials containing phages. Thus, biodegradable polymers, and principally water soluble polymers, have received more attention owing to their biodegradability and higher polarity, which contribute to the phage stability and viability [3]. In this work, three biodegradable films of sodium caseinate (SC), sodium alginate with gelatin (SAG), and polyvinyl alcohol (PVOH) were considered for preparing active films as carriers of Listex P100. It must be contemplated that the properties of these films can be modified by introducing bioactive compounds in their formulation and they can be easily applied as coating over other polymeric substrates [11]. To date, there are hardly any studies on the development of active films containing Listex P100 phage owing to the decrease in the growth of *L. monocytogenes*. Moreover, the application of sodium caseinate or sodium alginate with gelatin as carriers of different bacteriophages against others pathogen microorganisms is very rare, and in the case of PVOH polymers, it is inexistent [9,12]. PVOH is a synthetic biodegradable and biocompatible polymer, approved by the FDA for use in food contact and as a food additive and with excellent film-forming properties [13,14].

The use of bacteriophages to control pathogens is a hopeful alternative; nevertheless, its incorporation into active packaging is still unknown, so it is essential to research this field. Thus, the objective of this work was the study of the inhibitory and bactericidal capacity of Listex P100 bacteriophage against *L. monocytogenes* in different growth phases. In addition, different phage carrier matrices were developed and their antilisterial capacity and the effect of the incorporation of Listex P100 phage on the polymeric properties were analysed. The results showed that the incorporation of phages did not affect the colour, opacity, thermal stability, or morphology of the polymers, and they exhibited interesting antilisterial activities.

## 2. Materials and Methods

### 2.1. Biological and Chemical Agents

*Listeria monocytogenes* (CECT 934, ATCC 19114) pathogen strain was chosen for its impact on food-borne illness. The strain was maintained at −80 °C in a tryptone soy broth (TSB) supplemented with 20% glycerol. For experimental use, the stock culture was maintained by regular subculture at 4 °C on tryptone soy agar (TSA) and transferred monthly. Before use, a loopful of the strain was transferred to 10 mL of TSB and incubated at 37 °C for 24 h. All microbiological products were provided by Scharlab, Barcelona, Spain.

Bacteriophage preparation LISTEX™ P100 was obtained from MICREOS Food Safety Inc., Wageningen, The Netherlands. The bacteriophages were stored in the original suspension at 4 °C.

Polyvinyl alcohol (PVOH) was obtained from Gohsenol GH17, Nippon Synthetic Chemical Company, Osaka, Japan. Gelatin from porcine skin, type A, sodium caseinate (SC) (casein sodium salt from bovine milk), and sodium alginate (SA) were purchased from Sigma Aldrich (Madrid, Spain). Glycerol was obtained from Panreac Apllichem (Barcelona, Spain).

### 2.2. Antilisterial Activity of Listex P100

The inhibitory and bactericidal activities of Listex P100 bacteriophage (Bph) were studied against different concentrations of *Listeria monocytogenes* in stationary and exponential phases. A loopful of the *Listeria* stock culture was transferred to 10 mL of TSB and incubated at 37 °C for 24 h until stationary phase. Subsequently, serial dilutions with peptone water were made to obtain the following concentrations: 10^6^, 10^5^, 10^4^, and 10^3^ CFU/mL in TSB. These *L. monocytogenes* concentrations were put in contact with 10^8^ PFU/mL of Listex P100 and incubated for 0, 1, 6, 3, 24, and 48 h at 37 °C.

On the other hand, the overnight culture with an optical density of 0.9 at 600 nm was diluted in TSB and incubated at 37 °C until the exponential phase that corresponded to an optical density of 0.2 at 600 nm (10^5^ CFU/mL). Then, 100 µL of this concentration was inoculated in 10 mL of TSB in contact with 10^8^ PFU/mL of Listex P100 and incubated for 48 h at 37 °C.

Control samples without phages were prepared in each experiment.

After incubation time, samples were spread on selective medium Palcam agar Listeria (Scharlab, Barcelona, Spain) and incubated for 48 h at 37 °C. Colony forming units were counted and the results were expressed as log CFU/mL. Curves of microbial growth were plotted as a function of time, comparing the control and phage samples.

### 2.3. Preparation of Antilisterial Biodegradable Films

Sodium caseinate (SC), sodium alginate mixed with gelatin (SAG), and polyvinyl alcohol (PVOH) films were prepared by the casting technique based on the extension and evaporation of aqueous polymeric solutions at room temperature in sterile conditions. For this purpose, sodium caseinate was dissolved in distilled water at 4% with 31.25% of glycerol with respect to polymer content as plasticizer. Alginate sodium films were prepared at 1% with 40% of gelatin and 10% of glycerol respect polymer content using distilled water as solvent. PVOH was dissolved in distilled water at 2% with 10% of glycerol with respect to polymer content.

The mix solutions were heated (90 °C) and stirred for 30 min using a magnetic stirrer until completely dissolution and homogenization. The obtained film forming solutions (FFSs) were cooled and Listex P100 phage (Bph) was added (2 × 10^9^ PFU/15 g FFS). Subsequently, 15 g of each FFS was casted and dried for 24–48 h under laminar flow at room temperature, resulting in SC-Bph, SAG-Bph, and PVOH-Bph films. Control films without antimicrobial agent were also prepared (SC, SAG, and PVOH films).

Film thickness was measured with a digital micrometer (Mitutoyo Manufacturing Co., Ltd., Tokyo, Japan) with a sensitivity of 1 μm. Five readings were taken randomly for each film sample.

### 2.4. Morphological Characterization

The morphology of the film surface was observed by scanning electron microscopy (SEM) with a HITACHI S-4100 unit equipped with a BSE AUTRATA detector and an EMIP 3.0 image capture system (HITACHI, Madrid, Spain). Prior to analysis, films were cooled down to liquid nitrogen temperature and then broken in order to study the cross-sections. Samples were mounted on aluminium stubs using carbon adhesive tape and coated under vacuum with gold–palladium in a sputter coating unit. Images were captured at 5 kV.

### 2.5. Characterization of Optical and Structural Properties

#### 2.5.1. Optical Characterization: Colour and Transparency

The colour of control and phage-containing films was determined with a Konica Minolta CM-35000d spectrophotometer set to D65 illuminant/10° observer. The film specimen was placed on the surface of a standard white plate, and the CIELAB colour space was used to determine the parameters L* (lightness), a* (green/red), and b* (blue/yellow). The total colour difference ∆E* between films with and without phages was determined through Equation (1):

ΔE* = [(ΔL*)^2^ + (Δa*)^2^ + (Δb*)^2^]^1/2^ (1)

This parameter allows analyzing the effect of the incorporation of the phages on the color and visual appearance of the films. Analyses were carried out in triplicate. Five measurements were taken of each sample, and three samples of each film were measured.

Films opacity measurements were also performed according to the method of Han and Floros, 1997 [15,16]. Films were cut into rectangular shapes (9 mm × 30 mm) and placed inside the spectrophotometer cell at 600 nm. Five replicates of each film were tested. The opacity of the films was calculated following Equation (2):

O = Abs_600_/δ (2)

where O is the opacity index, Abs_600_ is the value of absorbance at 600 nm, and δ is the film thickness (mm).

#### 2.5.2. Fourier Transform Infrared (FTIR)–Attenuated Total Reflectance (ATR) Spectroscopy

FTIR–ATR spectroscopy was used to characterize the presence of specific chemical groups in the materials. FTIR spectra were performed in ATR mode with a Bruker IFS 66V spectrometer. The spectra were the results of 64 co-added interferograms at 4 cm^−1^ and resolutions in the wavenumber range from 4000 to 400 cm^−1^. The spectra analyses were performed using OPUS Software Version 7.

### 2.6. Thermal Properties of Antilisterial Films

The effect of phage incorporation on the thermal properties of alginate, caseinate, and PVOH polymers was analysed. The thermal stability of antilisterial biodegradable films was studied through thermogravimetric analysis (TGA) carried out in a Stare TGA system (Mettler Toledo GC20, Switzerland). Then, 5–6 mg of each sample was placed into alumina capsules and heated from 30 to 600 °C at a 10 °C/min heating rate under nitrogen atmosphere.

### 2.7. Antilisterial Activity of Films Containing Listex P100

The antibacterial capacity of developed films was tested against *L. monocytogenes.* Here, 10^3^ CFU/mL of *L. monocytogenes* was inoculated in 10 mL of TSB in contact with each film and incubated at 30 °C, which is the optimum temperature of phages. Samples were analysed after 1, 3, and 24 h of contact. Serial dilutions with peptone water of each sample were plated in Petri dishes with 15 mL of Palcam agar and incubated for 48 h at 37 °C. Colony forming units was counted and the results were expressed as log CFU/mL. The logarithmic reduction value (LRV) was calculated following Equation (3):

LRV = Control film (log CFU/mL) − Active film (log CFU/mL)(3)

The antilisterial film with best performance was chosen to study the antibacterial activity during storage at refrigeration temperature (8 °C). For this purpose, 10^3^ CFU/mL of *L. monocytogenes* was inoculated in 10 mL of TSB in contact with PVOH films with (active films) or without phages (control films). The tubes were stored at 8 °C and the samples were analysed at different times: 0, 1, 2, 5, and 8 days. Serial dilutions with peptone water of each sample were plated in Petri dishes with 15 mL of Palcam agar and incubated for 48 h at 37 °C. Colony forming units was counted and the results were expressed as log CFU/mL.

The experiments were carried out in triplicate.

### 2.8. Statistical Analysis

One-way analyses of variance were carried out. The SPSS computer program (SPSS Inc., Chicago, IL) was used. Differences in pairs of mean values were evaluated by the Tukey b test for a confidence interval of 95%. Data were represented as the average ± standard deviation.

## 3. Results and Discussion

### 3.1. Morphological Analyses of Developed Films

**Figure 1:**
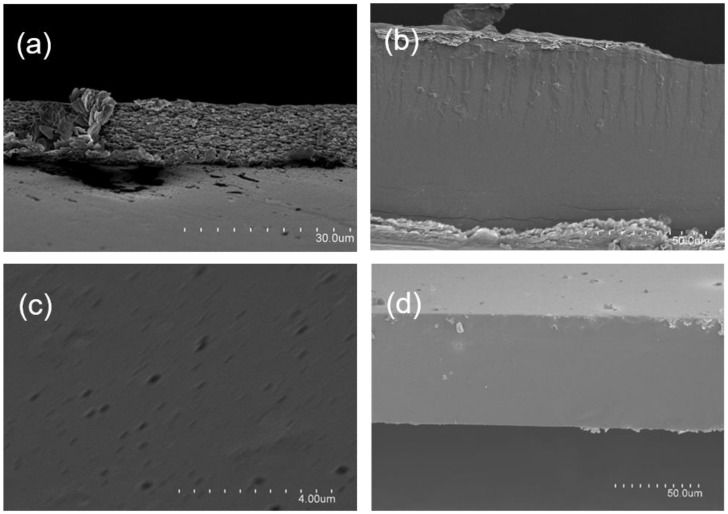
Scanning electron microscopy (SEM) images of cross-sections of sodium alginate mixed with gelatin (**a**), sodium caseinate (**b**) and (**c**), and polyvinyl alcohol (PVOH) (**d**) films carrying Listex bacteriophages.

The morphology of films was examined by SEM. Figure 1 shows the topography of the cross-section of films embedded with bacteriophages Listex P100. The surface and cross-section surface of the control films devoid of phages (results not shown) were very similar to the active films. All of them presented a uniform surface, a rough cross-section in SAG films (Figure 1a), and a soft surface in sodium caseinate (Figure 1b) and PVOH films (Figure 1d) without apparent phase separation owing to the addition of phages, indicative of good compatibility. Therefore, the incorporation of phages did not change the morphology of the films. Similar results were obtained for sodium alginate films embedded with Salmonella phage φ135 and E. coli phage vB_EcoS-EC4 [9].

In the case of control and active sodium alginate films, their cross-section manifested a uniform morphology with a high degree of roughness. SAG films were formulated with 40% of gelatin. This topography could be attributable to both polymeric phases and interphases separation of the polymer blend, suggesting a structural reorganization of polymer chains. It must be pointed out that, despite the addition of glycerol, sodium alginate films behaved in a brittle manner, which may also be indicative of a separation of phases of both polymers.

Figure 1c shows an enlarged image of the same area as Figure 1b. In this image, some “discontinuities” that break the smooth surface of the sodium caseinate films with a size of 250–350 nm were observed, which correspond to the expected size for Listex phages. Thus, it seems that phages were homogeneously distributed in sodium caseinate films.

### 3.2. Optical Properties of Antilisterial Biodegradable Films

Active and control sodium alginate mixed with gelatin (SAG), sodium caseinate (SC), and PVOH films was successfully obtained. All films were homogeneous and without discontinuities to the naked eye. The thickness, colour parameters, and opacity of the films with and without phages are shown in Table 1. The film thickness and opacity results showed significant differences between polymers principally owing to differences of polymeric concentrations, but the incorporation of phages did not affect these results. All films were quite transparent, accompanied by high lightness parameter (L*) values. In general, the transparency and colour of films or coatings are important factors that influence the overall appearance and quality of materials for food packaging applications. Excepting PVOH films that were colourless, SC and SAG presented a yellowish colour, indicated by increased negative a* values (green) and large positive b* values (yellow). Although ΔE values showed a total difference on colour between control and active films, they were not appreciated through visual appearance.

### 3.3. FTIR Spectra Results

**Figure 2:**
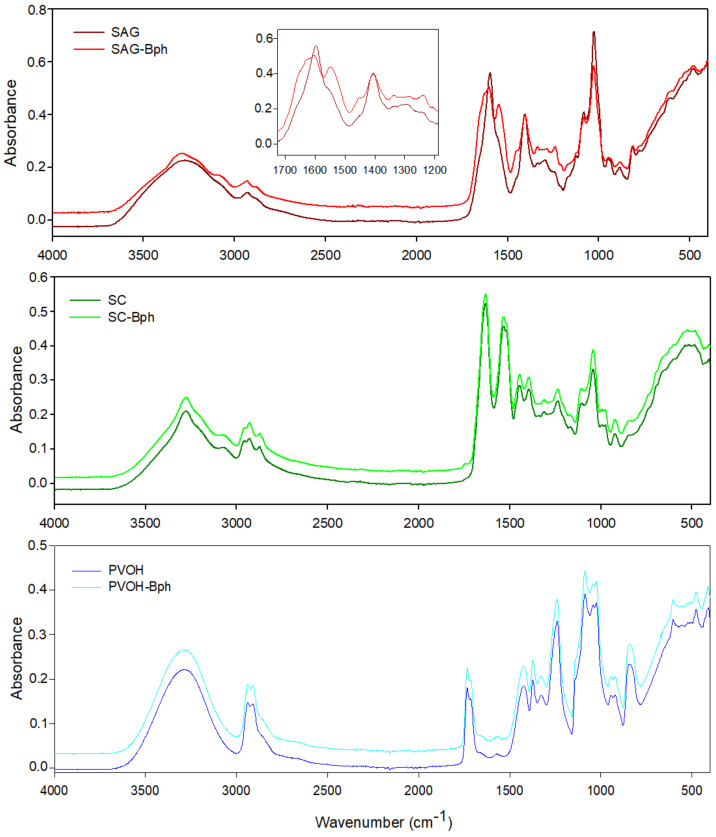
Fourier transform infrared (FTIR)/attenuated total reflectance (ATR) spectroscopy infrared spectra of developed control and antilisterial films. PVOH, polyvinyl alcohol; SAG, sodium alginate with gelatin; SC, sodium caseinate.

Structural analysis of films through FTIR analysis was used to study the occurrence of intermolecular interactions between the biopolymers and the bacteriophage Listex P100. The analysis of possible interactions between functional groups of polymers with their embedded phages through FTIR analysis has been very rare. In this study, sodium alginate was the unique polymer that showed some chemical interaction with Listex P100. As Figure 2 shows, both control and active SC and PVOH biofilms presented the same FTIR pattern spectra. Perhaps, this was because of the low intermolecular interaction between the phage and the chemical structure of polymers.

SC films exhibited the following characteristics bands: (1) a broad band with a peak at 3300 cm^−1^ associated with the hydroxyl stretching vibration, followed by a shoulder at 3070 cm^−1^ related to amine bonds; (2) peaks at 2927 and 2957 cm^−1^ assigned to CH stretching regions of CH_2_ and CH_3_ groups; (3) a peak at 2871 cm^−1^ from vibration of tertiary CH; (4) peaks at 1633 and 1534 cm^−1^ assigned to the amides I and II bands corresponding to the stretching of carbonyl group and to the symmetric stretching of N–C=O bond, respectively; and (5) a peak at 1236 cm^−1^ derived from the vibration of the amide bond in the plane of C–N and N–H [17,18,19]. On the other hand, the most relevant features of the PVOH spectrum were the wide hydroxyl stretching bands between 3000 and 3600 cm^−1^; bands at 2938 and 2908 cm^−1^ associated with the asymmetrical and symmetrical CH stretching bands, respectively; vibration of non-hydrolyzed acetate groups at 1733 and 1714 cm^−1^; the secondary hydroxyl group vibration in-plane bending band (1422 cm^−1^); the C–H wagging vibrations band (1327 cm^−1^); and the C–O stretching band at 1087 cm^−1^ [13,20].

Unlike the SC and PVOH polymers, gelatin-reinforced alginate films displayed certain band shifts and a different spectral pattern principally in the range between 1500 and 1725 cm^−1^ (see insert Figure 2), derived from the interactions between SAG functional groups and the phages. Control SAG film exhibited a peak at 1596 and 1408 cm^−1^ (asymmetric and symmetric stretching vibration of C–O bond of COO^−^ group, respectively), with a shoulder at 1551 cm^−1^ related to vibration of carboxylate groups [21] and N-H bending from gelatin [22], while SAG-containing phages film spectra evidenced a broader peak at 1601 cm^−1^, and the second peak was intensified and displaced to 1549 cm^−1^. These changes were unique for this active biopolymer and because some intermolecular interactions between SAG carboxylate and amine groups and phages were displayed. Other characteristic peaks common of SAG were the broad band for hydroxyl group stretching (from alginate) and N-H stretching of secondary amide (from gelatin) between 3000 and 3600 cm^−1^, as well as bands at 2930 and 2878 cm^−1^ associated with asymmetric and symmetric stretching modes of CH_2_ and CH, respectively [23,24,25]. Alves et al. (2020) have also studied sodium alginate emulsion-based films containing a phage cocktail and cinnamaldehyde through FTIR, and changes observed between 3000 and 3600 cm^−1^ were reproached owing to interactions between alginate and cinnamaldehyde [12].

### 3.4. Thermal Properties

**Figure 3:**
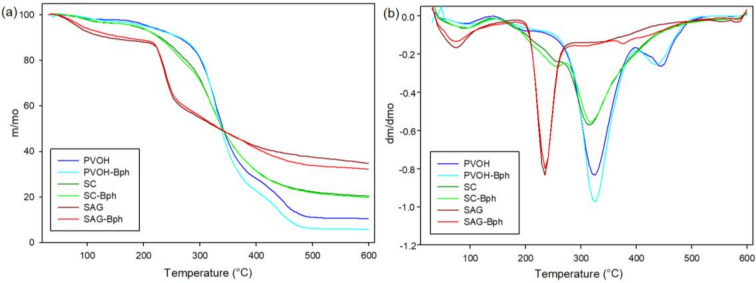
Thermogravimetric analysis (TGA) (**a**) and derivative TGA (DTGA) (**b**) curves of developed control and active films.

Figure 3 presents the TGA and their derivative TGA (DTGA) curves of developed films with the principal purpose of evidencing the effect of incorporated Listex P100 on the thermal stability of polymers. Thermal decomposition curves of control and containing-bacteriophages films presented a similar profile and temperatures of onset and maximum degradations (see Table 2). The presence of phages did not accelerate or affect the processes of polymeric thermal degradations. TGA thermograms revealed an initial mass weight between 60 and 150 °C regarding the evaporation of free and associated water due to the hydrophilic character of polymers. Subsequently, PVOH evidenced two degradation processes associated with the separation of lateral groups resulting on acetic acid, water, and acetaldehyde by-products (approximately 327 °C) and the decomposition of the main PVOH polymeric chain (~446 °C). Some authors have declared that the principal decomposition process is associated with the crystalline polymeric section, whereas the continuing shoulder is related to the molten state [26,27].

On the other hand, SAG films displayed a single degradation stage between 220 °C (T_onset_) and 470 °C (approximately 46% weight loss) related to the decomposition of glycosidic bonds and hydroxyl groups. Other studies have also manifested that degradation of polysaccharides starts at around 200 °C, accompanied by a random split of the glycosidic bonds, vaporization, and elimination of volatile compounds [23,28].

SC films presented a decomposition process of caseinate constituents with a maximum degradation at approximately 320 °C. This protein thermal degradation was previously accompanied by a shoulder (T_deg1_ ~ 258 °C) that could be associated with the loss of glycerol, as SC films presented the highest concentration of this plasticizer (31.25 wt% with respect to polymer content) [17]. Table 2 also indicates the residue (% initial weight) of films at 500 °C. Both caseinate and SAG films presented high residual chars. Some authors have declared that this fact is typical in protein materials because the high polarity difference between skeletal amide and hydrocarbon side chains produces high intermolecular hydrogen-bonded and amide–amide interactions, which promotes segregation and char formation during heating processes [29].

### 3.5. Antilisterial Activity of Listex P100

**Figure 4:**
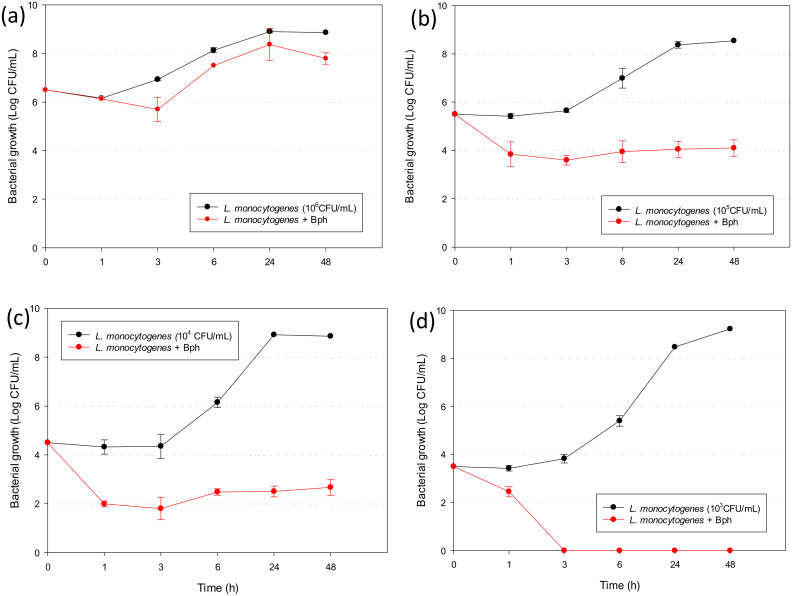
Antilisterial activity of Listex P100 against (**a**) 10^6^ CFU/mL of pathogen; (**b**) 10^5^ CFU/mL of pathogen; (**c**) 10^4^ CFU/mL of pathogen; and (**d**) 10^3^ CFU/mL of pathogen. Mean values and 95% LSD intervals.

The inhibitory and bactericidal activities of Listex P100 phage were studied against different concentrations of *Listeria monocytogenes* in stationary and exponential growth phases. As can be seen in Figure 4, the phage presented a high antimicrobial activity when the microorganism was in stationary phase with all concentrations tested. Control samples without phage evidenced growth until reaching around 9 log after incubation at 37 °C and 24 h independently of the initial inoculum. This value was maintained during 48 h, when microorganism again reached the stationary phase and started to decrease slowly in the death phase. The antilisterial effect was dependent on the initial inoculum of pathogen. When the initial inoculum was 10^6^ CFU/mL, the phage suspension was able to be reduced by 1.20 log after 3 h and the antilisterial effect was maintained over time (Figure 4a). In the presence of 10^5^ and 10^4^ CFU/mL, the inhibition was already observed in the first hours and the antilisterial effect obtained was 4.40 and 6.19 log after 48 h, respectively (Figure 4b,c).

Finally, bactericide activity was obtained when the initial bacterial inoculum was reduced to 10^3^ CFU/mL (Figure 4d). When the number of bacteria present in the liquid medium is low, the phage–pathogen interaction for that bactericide effect was not observed until three hours.

In the exponential phase, the suspension phage was reduced by 4.53 log after 24 h (data not shown). Microorganisms in the exponential phase are usually more sensitive to antimicrobial agents owing to its active growth; however, in the stationary phase, they are more resistant. Genetic expression and charge surface change during the growth phases of microorganisms, making the antimicrobial–bacteria interaction along the exponential phase more accessible [30,31]. Studies reported with several broad spectrum antibiotics produced 99.9% killing when the bacteria was in optimal growth, and the effectiveness was largely dependent on the growth rate [32].

In our work, the antimicrobial effect was high and similar regardless of the growth phase, but it must be considered that the studies with microorganisms in stationary phase are more relevant owing to the difficulty in killing them in those growth conditions. The effectiveness of phage was successful against all concentrations tested, but the activity increased as the initial inoculum of pathogen decreased. At a high concentration (Figure 4a), the antimicrobial effect was bacteriostatic, producing an increase in the lag phase, and the growth curve was below the control curve; however, when the concentration was increased, the effect produced was inhibitory (Figure 4b,c) and bactericide (Figure 4d). As phages are nonmotile, the antilisterial effect is dependent on the concentrations used [33]. Anyway, phage particles were able to find the host bacteria in the liquid medium and produce new cycles of infection [10]. On the other hand, the phage concentration was fixed according to previous reports that showed efficacy between 2 × 10^7^ and 2 × 10^8^ [33].

### 3.6. Antilisterial Activity of Films Containing Listex P100

**Figure 5:**
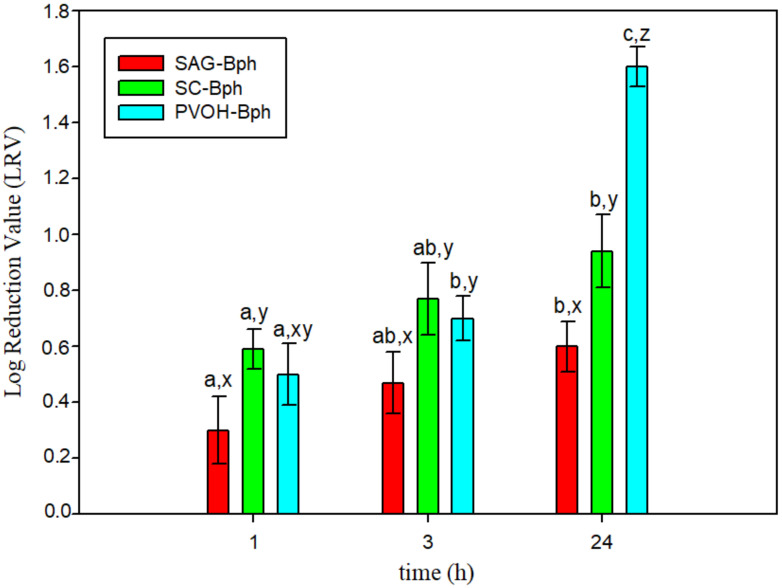
Log reduction values (LRVs) of active films at different times (**a**–**c**) indicate differences of LRV of the same film at different times. (**x**–**z**) indicate differences between films at the same time.

The antibacterial capacity of developed films was tested against *L. monocytogenes* after 1, 3, and 24 h of contact. The results as log reduction values (LRVs) of phage are shown in Figure 5.

Films developed presented antimicrobial activity against *L. monocytogenes* and the inhibition increased with time. The results indicated that phages are released into the liquid medium during the first 24 h, and the viral multiplication process still occurs owing to new virus particles being released and infecting new bacteria [10]. SAG-Bph films showed the lowest antimicrobial effect, with 0.60 log reduction after 24 h. Inhibition of growth obtained with SC-Bph and PVOH-Bph was similar during the first 3 h, but the effect of PVOH-Bph films was greater than SC-Bph after 24 h. This fact was probably because the phages were more retained in PVOH films and its release and interaction with bacteria was slower.

The application of free phage showed high antimicrobial activity, but their immobilization in different matrices can decrease their viability or availability [3,5]. Other authors obtained similar results; Lone et al. (2018) showed a minor effectiveness of immobilized phages on cellulose membranes compared with the application of free phage suspension against *L. monocytogenes* growth in contaminated cantaloupe [3]. Radford et al. (2017) immobilized bacteriophages to a xanthan coating applied on poly-lactic acid, which reduced *Salmonella* Typhimurium and *L. monocytogenes* growth on precooked sliced turkey breast combined with vacuum packaging; their results showed that *L. monocytogenes* counts decrease 2 logs in the presence of phages [34].

Previous studies have shown that the incorporation of phages into polymeric matrices may produce a loss of their activity over time [35,36]. Phage stability depends on the matrix on which it was applied. The loss of activity with films compared with phage free can be attributed to the incorporation in the matrix, where phages are exposed to stress conditions such as mixing, agitation, desiccation, and drying [36]. Leverentz et al. showed that the application of phage P100 on apple slices was less antilisterial than on melon slices as a result of lower pH [37,38]. Oliveira et al. (2014) determined that phage treatment was more effective on fresh-cut melon then on pears for the same reason [39]. The pH of the film forming solutions was measured before casting: SAG (pH 5.78), SC (pH 6.50), and PVOH (pH 6.90). During the casting method, the solvent evaporates slowly, during 24–48 h, and the compounds of the film are concentrated. The lower pH of SAG film forming solution could produce an acidic environment during this process and, consequently, decrease phage viability. The optimum pH of Listex P100 is 7, which could be related to the greater effectiveness observed with PVOH films [40].

**Figure 6:**
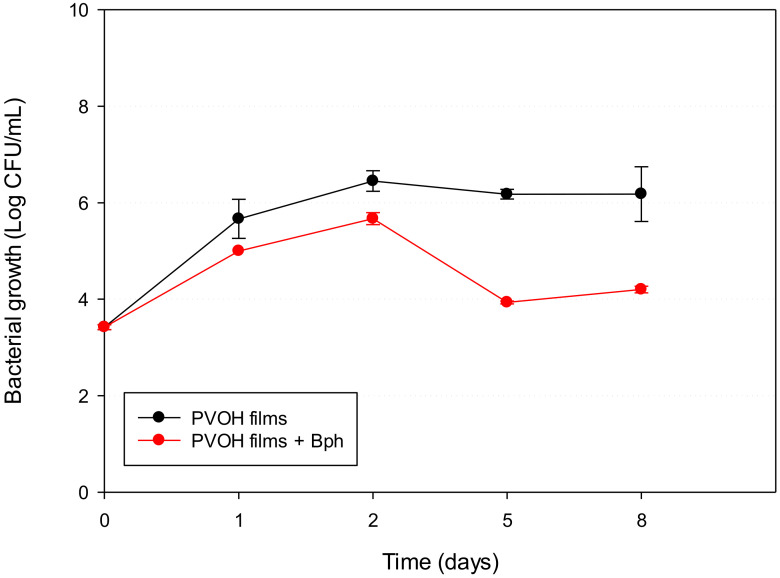
Antilisterial activity of PVOH films at 8 °C during 8 days.

Because PVOH-Bph was the active material that presented the best antilisterial performance, this film was chosen to study the antibacterial activity during storage at refrigeration temperature, and the samples were analysed for 8 days. As can be seen in Figure 6, the antimicrobial effect was initially bacteriostatic owing to the slow release of phage and, after 5 days of storage, the effectiveness increased, producing an inhibitory effect with reductions of around 2 log at the end of the storage period.

The optimum temperature of phages is 30 °C; for this, higher temperatures of post-phage treatment can produce a greater percentage reduction of the pathogen bacteria; however, because of the shorter generation of *L. monocytogenes* at this temperature, the microbial counts are also greater [41]. Our results showed that the effectiveness of PVOH-Bph films was higher at refrigeration temperature, because *L. monocytogenes* is a psychrotropic bacterium, which means that they are able to grow at low temperatures, but slowly improve the phage activity. On the other hand, the obtained results indicated the high viability of phage maintained their effectiveness over 8 days. The log reduction value obtained indicated success because the *Listeria* concentration used in this experiment was excessive compared with that of natural food products. Regulations of the presence of *L. monocytogenes* depend on local authority rules. In the USA, it is the absence in 5 × 25 g of food as well as in the environment of food processing, and the European Union legislations require absence in 10 × 25 g in children food or 5 × 25 g in ready to eat foods during the manufacture, and it may not exceed 100 CFU/g during the shelf life [41,42,43].

There is a requirement for the development of alternative materials to replace conventional plastics, which are non-biodegradable materials and derive from fossil and non-renovable sources [44]. In this work, biodegradable antimicrobial films with water-soluble properties have shown compatibility with the phage suspension. This fact is relevant because active packaging design must consider the compatibility between the active agents and polymeric matrices in order to obtain homogenous materials and allow their release [45]. These matrices are not only derived from natural sources, but they are safe and do not generate negative environmental impact. On the other hand, phages are part of habitual microbiota of eukaryotic organisms and are usually consumed in fermented foods and are eliminated with the stomach acidity and digestive proteinases [40]. Furthermore, toxicity studies have demostrated that Listex P100 is not dangerous to human health and it is strictly lytic to the bacteria population. Although no reports of environmental impacts are available, the probability of persistence of Listex P100 in the environment is very low as the population of bacteriophage naturally declines over time without a susceptible host [40].

## 4. Conclusions

Antimicrobial activities of bacteriophage Listex P100 against different initial concentrations of *Listeria monocytogenes* were confirmed. Listex P100 was successfully incorporated into gelatin-reinforced alginate, sodium caseinate, and polyvinyl alcohol matrixes to develop antilisterial active materials. Physical characterization of active materials by means of morphological, optical, structural, and thermal analysis indicated that the incorporation of phages did not negatively affect polymeric materials. Besides, all active biodegradable films presented increasing antilisterial activity over time, and polyvinyl alcohol-based material exhibited the best performance. In conclusion, the developed films could be applied in packaging material for additional control of pathogens in combination with hurdle technologies to ensure safer food products.

## Figures and Tables

**Table 1:** Thickness (µm), colour parameters, and opacity of films with and without Listex P100. PVOH, polyvinyl alcohol; SAG, sodium alginate with gelatin; SC, sodium caseinate.

Films	Thickness	Opacity	L*	a*	b*	ΔE
SAG	31.3 ± 1.9 ^a^	1.9 ± 0.2 ^c^	89.4 ± 0.1 ^b^	−0.48 ± 0.02 ^b^	2.07 ± 0.32 ^a^	-
SAG-Bph	32.0 ± 1.8 ^a^	1.8 ± 0.2 ^c^	89.3 ± 0.5 ^b^	−0.52 ± 0.02 ^a^	2.17 ± 0.03 ^a^	0.56 ± 0.01 ^b^
SC	91.7 ± 1.7 ^c^	0.6 ± 0.1 ^a^	89.8 ± 0.2 ^c^	−0.38 ± 0.01 ^c^	1.75 ± 0.05 ^d^	-
SC-Bph	92.3 ± 1.6 ^c^	0.7 ± 0.1 ^a^	88.0 ± 0.1 ^a^	−0.34 ± 0.01 ^d^	1.69 ± 0.02 ^c^	1.86 ± 0.14 ^c^
PVOH	48.0 ± 1.9 ^b^	1.2 ± 0.1 ^b^	89.0 ± 0.8 ^b^	−0.14 ± 0.02 ^e^	−0.04 ± 0.03 ^d^	-
PVOH-Bph	48.6 ± 1.8 ^b^	1.1 ± 0.3 ^b^	89.1 ± 0.1 ^b^	−0.15 ± 0.01 ^e^	−0.01 ± 0.04 ^c^	0.08 ± 0.02 ^a^

Different superscripts a–e within the same column indicate significant differences among the values of a parameter between different films according to analysis of variance (ANOVA).

**Table 2:** Thermal parameters of degradation processes of developed films.

Films	T_onset_ (°C)	T_deg1_ (°C)	T_deg2_ (°C)	W_500°C_ (%)
SAG	223.3	236.9		37.5
SAG-Bph	222.6	238.1		33.9
SC	201.8	258.0	316.9	22.4
SC-Bph	206.5	261.1	320.2	22.5
PVOH	285.3	327.7	446.3	11.1
PVOH-Bph	292.6	328.2	437.7	6.2
